# Synthesis and Preparation of (Acrylic Copolymer) Ternary System Peelable Sealing Decontamination Material

**DOI:** 10.3390/polym12071556

**Published:** 2020-07-14

**Authors:** Zhiyu He, Yintao Li, Zhiqiang Xiao, Huan Jiang, Yuanlin Zhou, Deli Luo

**Affiliations:** 1 State Key Laboratory of Environment-Friendly Energy Materials, School of Materials Science and Engineering, Southwest University of Science and Technology, Mianyang 621010, China; hezhiyu@swust.edu.cn (Z.H.); liyintao@swust.edu.cn (Y.L.); jhuan0727@163.com (H.J.); 2 Communist Youth League of Southwest University of Science and Technology, Mianyang 621010, China; 3 Institute of Fluid Physics, China Academy of Engineering Physics, Mianyang 621010, China; xzhiqiang@21cn.com; 4 Institute of Fluid Physics, China Academy of Engineering Physics, Jiangyou 621908, China

**Keywords:** stripping, enclosed, decontaminant

## Abstract

Traditional methods that are used to deal with radioactive surface contamination, which are time-consuming and expensive. As one effective measure of radioactive material purification, strippable coating, which effectively coats the pollutant, and settles them on the surface of objects. However, there are some shortcomings in terms of film formation and peelability, such as a brittle coating and poor peelability. Therefore, in order to meet the treatment methods for radioactive contaminants needs, the strippable coating must have excellent sealing, corrosion resistance, weather resistance, low environmental pollution, short film formation time, and good mechanical properties; in addition, the spraying process should be simple, with moderate adhesion, and it should be capable of being quickly and completely peeled off. In this paper, a ternary system was prepared by pre-emulsion polymerization with butyl-acrylate, methyl methacrylate, acrylic acid as the reactive monomer, sodium dodecyl sulfate as the active agent, potassium persulfate as the initiator, and water as the dispersion medium. The Fourier-transform infrared (FTIR) spectroscopy, nuclear magnetic resonance (^1^H-NMR), ICP emission spectrometer, surface tension tester, and universal testing machine were used to characterize the structure and morphology of the composite materials. The results show that the decontaminant can quickly wet the powder particles and the surface pollutants. The sealing efficiency of Fe and Cu was over 90%. After the decontaminant was cured, it could be continuously formed on the surface of different substrates and be completely peeled off, as well as having excellent film formation and peelability.

## 1. Introduction

Upon the retirement of nuclear facilities, hazards include the large number of radioactive surface contaminations generated. When the large surface contaminants are moved or transported or even decontaminated, they can cause the surface discharge of pollutants into the atmosphere, leading to the generation of radioactive aerosols, creating environmental problems [1]. Therefore, in the process of the decontamination of nuclear facilities, it is necessary to find a kind of decontamination agent that can effectively coat the pollutants on the surface of nuclear facilities. It needs to not only effectively coat the pollutants on the surface of the equipment, but must also play a fixed and isolated role in preventing the generation of secondary wastes.

At present, the treatment methods for radioactive contaminants on the surface of equipment mainly include cleaning, heating, chemical, electrochemical, and mechanical cleaning methods [2,3,4,5].

At the same time, the treatment methods for radioactive contaminants are generally divided into physical methods and chemical methods. Such as mechanical decontamination, sandblasting, ultrasonic decontamination, washing, decontamination [6,7,8].and reagent washing, electrochemical gel decontamination, and self-brittle film peeling [9,10,11,12]. The common problem with the existing decontamination methods is that secondary pollution occurs easily. For example, because produced a lot of radioactive contaminated water [13,14,15,16]. the application of high-pressure jet cleaning and the use of ammonium salt for ion cleaning to promote the exchange of radioactive cesium ions have been limited. Therefore, the surface purification of radioactive materials is an urgent task of great significance. As one of the effective measures of radioactive material purification, a strippable solid seal coating has been developed [17,18,19] to effectively enrich surface contaminants during the formation of the coating. At the same time, it can effectively capture suspended particles in the air; enrich and settle them on the surface of objects; and finally, achieve the purpose of capturing, enriching, stripping, and deconsolidating surface pollutants.

In the study of Sun et al. [20], By using the P-DAP prepolymer as the main film-forming material, TPGDA as the active diluent, and bis (2,4,6-trimethylbenzoyl) phenylphosphorous oxide (BAPO) as the light initiator, a new type of UV-curable strippable coating was prepared. William M.M. et al. [21] using the emulsion polymerization method, prepared water-based peel emulsion decontamination coatings, which exhibited an excellent protective effect and were easy to peel off. Gertzmann Shan et al. [22] prepared a water-based peel coating with acrylic polymer emulsion—the coatings had good water resistance, protective effects, and adhesion. R. et al. [23] used acrylate and methacrylate to prepare water-based decontamination coatings by emulsion polymerization, which can be widely used on different surfaces. Zhou et al. [24] prepared self-brittle decontamination coatings by emulsion polymerization. Li et al. [25,26] reported that a type of gas phase antirust coating was prepared using vinyl polymer resin as the main film-forming material, adding a compound oil–soluble gas phase corrosion inhibitor, lubricating oil, mixed solvent and functional additives. Yin et al. [27] reported an efficient approach for the fabrication of a strippable coating through doped SO_4_^2−^/TiO_2_ into polyphenylamine;this coating had a good electrical conductivity, and it had good decontamination performance.

Existing strippable coatings have obvious problems in film forming and strippability, which are mainly manifested in the high brittleness of the coating and the poor strippability. As a result, their application is greatly restricted. Therefore, in order to keep the equipment in good condition at all times and to meet emergency needs, the future strippable decontaminant will surely have the following comprehensive performance [28,29,30,31]:

1. Short curing time, good physical properties, and friendly to the environment.
2. The film body formed by the coating has better elongation at break and tensile strength; it can adapt to different object surface.
3. The painting process is simple, the adhesion is moderate, it can be quickly and completely peeled off.
4. On the premise of meeting the requirements of decontamination, it is necessary to expand and study the protective performance of the decontaminant on the surface of objects.

Compared with other films, acrylate film has good transparency and glossiness, diversity of comonomers, and adjustability of the resin structure. However, the homopolymer properties of acrylate and methacrylate make it difficult to meet the requirements needed for coating film-forming materials; for example, polymethyl methacrylate, which is the raw material of plexiglass, is too brittle as a film-forming material, and polybutyl acrylate is too soft and sticky, and is not suitable for coatings. Therefore, as the film-forming material of the paint, acrylate copolymers are ideal, and are usually obtained by polymerizing different monomers [32,33,34].

Table 1 is the monomer commonly used in acrylic polymer emulsions. In the ternary copolymerization, two main monomers are often used to determine the basic properties of the synthetic substance, and then a small amount of a third monomer is added for special modification. In this paper, acrylic resins are used for the synthesis of peelable coating substrates. Most of the synthesis methods are emulsion polymerizations. Compared with other polymerization methods, emulsion polymerization has the following advantages [35]:

1. The temperature effect is small, the polymerization reaction can be carried out at a lower temperature at night, it has a higher polymerization rate, and the synthesized polymer has a higher molecular weight;
2. Using water as a medium, the reaction system has a large specific heat capacity, and the system viscosity is small and conducive to heat dissipation;
3. Emulsion products can be directly used as coatings, adhesives, and surface treatment agents; the synthesized substances are environmentally friendly and have less potential safety hazards.

With the development of technology, the preparation methods of polyacrylate emulsion are as follows: (1) batch emulsion polymerization, (2) semi-continuous emulsion polymerization, (3) continuous emulsion polymerization, and (4) seed emulsion polymerization. In terms of industrial production, more conventional emulsion polymerization is used, mainly because of technical difficulties, controllability of conditions, and production costs [36].

In order to get closer to the actual production, in this paper, pre-emulsion polymerization was used to synthesize the polyacrylate emulsion. Compared with the existing methods, pre-emulsion polymerization has the following characteristics and advantages [37,38,39]:

When the pre-emulsification process is used, compared with the non-pre-emulsification system, the monomer droplets formed by the monomers will not adsorb the emulsifier from the surroundings, which reduces the probability of gel formation and helps maintain the stability of the party system.

When the pre-emulsification process is used, the addition of an emulsifier and monomer is not completed in one addition and instead is added gradually. When the emulsifier and monomer are gradually added as the reaction proceeds, the monomer conversion rate can be improved and the particle size of the colloidal particles can be controlled.

When the pre-emulsification process is adopted, the functional monomers before the reaction are mixed by the pre-emulsification machine, which is conducive to the reaction between the monomers and makes the structure of the synthesized copolymer more uniform.

Therefore, in this paper, methyl methacrylate and butyl acrylate were used as reactive monomers. A pre-emulsification method was used to prepare a peelable coating. Methyl methacrylate can provide a certain hardness and adhesion to the membrane body and can improve the mechanical performance of the membrane. Butyl acrylate can adjust the flexibility of the film body, and can make the film body soft and hard, with a better peelability after curing it into a film. It was characterized by infrared and nuclear magnetic methods, and the results show that the target product was synthesized. Through the research on the film forming, peeling, and airtightness of the different substrate surfaces, the results show that the synthetic coating can form a continuous film and completely peel off on different surfaces, which can effectively prevent gas penetration. After curing and forming a film, the blocking rate of the iron and copper elements was greater than 90%.

## 2. Experiment

### 2.1. Chemicals

Acrylic acid (AA), Butyl propionate (BA), Methyl methacrylate (MMA), potassium persulfate, sodium dodecarboxybenzene sulfonate, and OP-10 were all analytically pure and were purchased from Kelong Chemical Reagent Co., Ltd. (Chengdu, China).

### 2.2. Synthesis

**Figure 1:**
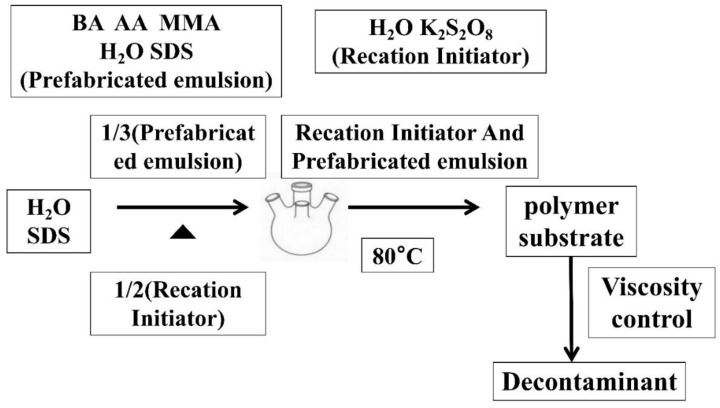
Process flow chart of decontaminant preparation.

Figure 1 is the Process flow chart of decontaminant preparation. Details are as follows:

(1) First, 5 g of OP-10 emulsifier was dissolved in 450 mL of distilled water, and was stirred and dissolved in a 30 °C water bath; 5 g of sodium dodecyl sulfate was dissolved in a clear solution containing an emulsifier, and was stirred until clear. Then, 500 mL of water was added, stirred well, and set aside; 6 g of potassium persulfate was added to the water and was dissolved.
(2) We added 100 g monomer, 100 mL emulsifier, and 100 mL distilled water into a beaker at room temperature, and used an emulsifying machine for the pre-emulsification of the fluid.
(3) We added the distilled water and emulsifier into four mouth flasks, each equipped with a reflux condensing tube thermometer with an agitator and a constant pressure drip funnel, then heated the water to 75 °C. Next, we added 100 mL of pre-emulsion and 50 mL of potassium persulfate solution, and the remaining pre-emulsion was preserved in a constant pressure funnel for later use.
(4) When the emulsion was blue, the remaining pre-emulsion and potassium persulfate solution were added synchronously and slowly, which lasted 2–3 h, and then the temperature was raised to 80 °C. After 2 h of insulation, the reaction ended. Next, cooling and discharging were completed.
(5) Then, the pH value was adjusted to the desired viscosity in order to obtain the polymer substrate.

### 2.3. Structural Characterization

The contact angle was tested using a surface tension tester. The temperature ranged from −10 to 130 °C, and the temperature control precision was 0.01 °C.

Mechanical spraying device: air compressor by Zhejiang Wuyi Guangli Mechanical and Electrical Co, Ltd. (Zhejiang, China).

Physical and mechanical properties of the coating: an electronic universal testing machine, namely Qj-210, from Shanghai QING JI instrument technology Co., Ltd. (Shanghai, China). The sample was 1 mm thick, the shape was a #4 dumbbell-shaped specimen, and the tensile speed was 200 mm/min^−1^.

FTIR and nuclear magnetic resonance (^1^H-NMR) characterization: the chemical structures of the samples were analyzed by Fourier-transform infrared (FTIR) spectroscopy (Nicolet 5700, (Thermo Fisher Scientific, Waltham, MA, USA) with a KBr sampling sheet.

The ^1^H-NMR spectrum of the powder samples was obtained using an Avanceow 600 spectrometer (Bruker, Karlsruhe, Germany), with D_2_O as the solvent.

Particle size analysis: the test was carried out using a Malvern 3000 HS nanoparticle (Malvern Instruments Ltd., Worcestershire, UK) size and potential analyzer.

Gel permeation chromatography: instrument model: Waters-201 GPC instrument; separation column type: Plgei10μMlXED-B×3 (Agilent Technologies Inc./HP, Palo Alto, CA, USA) high cross-linked spherical polystyrene-divinylbenzene polymer column; standard sample: polystyrene; tetrahydrofuran; flow rate: 1.000ml/min; detector: differential refractometer; temperature: 40 °C.

H_2_ permeability test: according to GB/T1308-2000, use the GDP-C permeability tester of German Brugger company (Munich, Germany), the permeation gas is H_2_, and the test temperature is 25 °C. The test result is the permeation rate of hydrogen when it permeates steadily, and has been converted to standard pressure and temperature.

### 2.4. Seal Performance Rate Test

Seven sets of symmetrical points were selected on the work surface, which were counted as left side 1, left side 2, right side 1, and right side 2, and convex 1, convex 2, and convex 3. Left side 1, left side 2, right side 1, and right side 2 were the ordinary surfaces of the spheres in the can body, which were grouped into one group; on the other hand, convex 1, convex 2, and convex 3 were classified as one group. The points above the seven test points were the base pollutant extraction points. The surface before treatment without the sealant was sampled at a sampling point of a predetermined area (10 cm × 10 cm), by using a cotton yarn impregnated with petroleum ether, and the cotton yarn was repeatedly rubbed and sampled while the background contaminant was extracted. The cotton yarn samples (14 pieces), sampled before and after the solid sealing, were immersed in a nitric acid solution that had a volume of 200 mL and a concentration of 5 mol/L, and were heated to 80 °C in a water bath for 1 h. After the solid matter on the cotton yarn was completely dissolved and the petroleum in the upper layer of the aqueous solution was completely volatilized, the pH of the solution was tested to ensure that it was about pH 4–5, which became the test sample solution after filtration. The treated solution passed through an ICP emission spectrometer, an inductively coupled plasma spectrometer, and the content of Fe and Cu in the solution was measured. The test of the seal performance rate is calculated using Formula 1.

(1) $$Seal\;Performance\;Rate=1-\;\frac{Metal\;Ion\;Content\;After\;Sealing}{Metal\;Ion\;Content\;Before\;Sealing}$$

## 3. Results and Discussion

**Figure 2:**
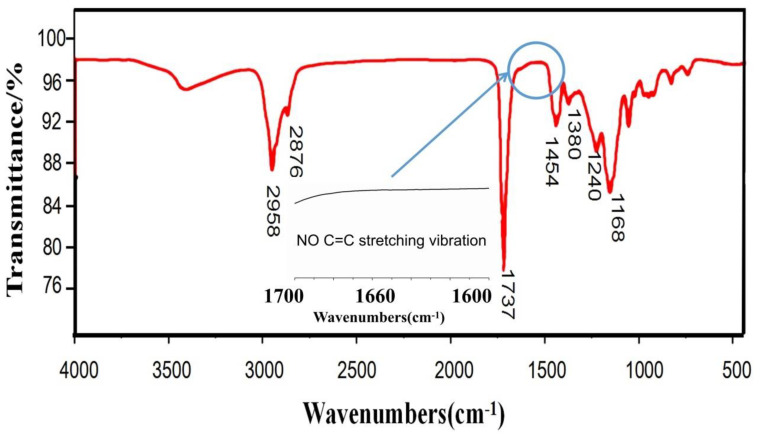
The infrared spectra of polymer.

**Figure 3:**
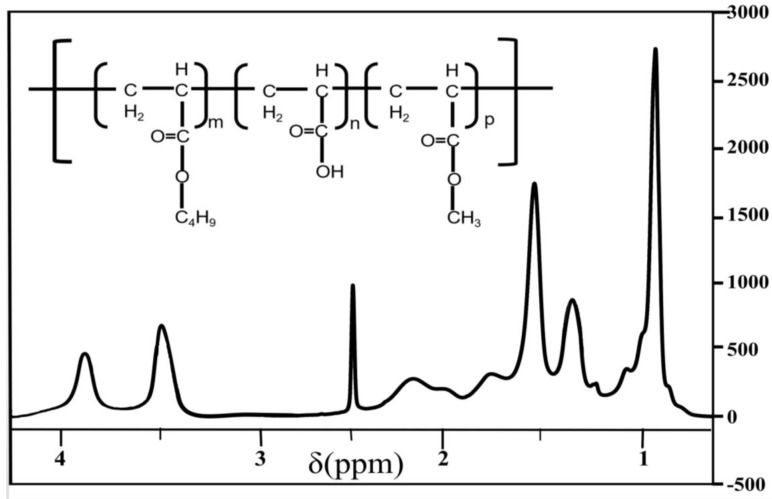
NMR spectra of polymer.

**Figure 4:**
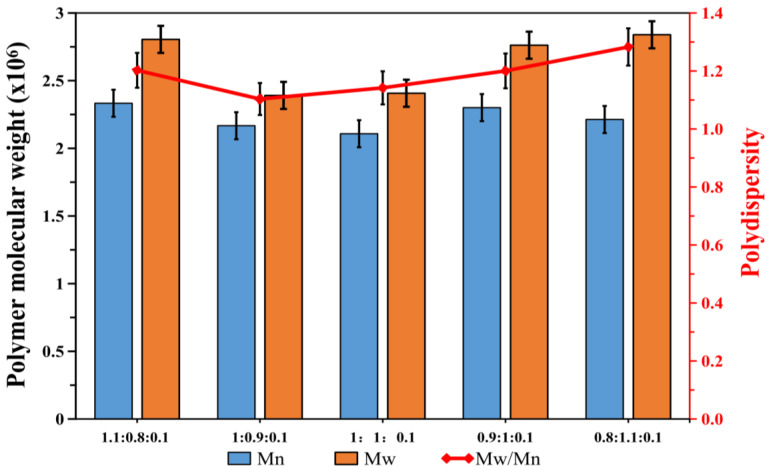
The GPC (Gel Permeation Chromatography) test of different monomer ratios (BA/MMA/AA).

Figure 2 displays the FTIR spectra of the polymer. It can be found that the O-H stretching vibration of the hydroxyl groups was confirmed by the band at 3418 cm^−1^. The bands at 2958 and 2876 cm^−1^ resulted from the -CH_3_ and -CH_2_- stretching vibrations, respectively. The C=O stretch of the butyl acrylate and methyl methacrylate was confirmed by the bands at 1737 and 1731 cm^−1^, respectively. The C-O stretching vibration of the carbon–oxygen bond of ester groups was confirmed by the bands at 1454 and 1168 cm^−1^. As shown in the magnified 1700–1600 cm^−1^ infrared spectrum diagram, there were no characteristic C=C bond absorption peaks in the regions of 1690 and 1620 cm^−1^. The results indicate that the reaction occurred successfully in the monomers. In addition, the ^1^H-NMR spectra of the polymer are shown in Figure 3. For the calculation of the number of H bonds in the polymer, the area under the peak (3.6 ppm (h)) corresponding to the CH_2_-CHCH_2_ bonds was used for normalization to a constant of 1. In the ^1^H-NMR test results, the chemical shift at 0.96 ppm was mainly attributed to the absorption peak of H in the methylene hydrogen of CH_3_(CH_2_)_3_OOC; the chemical shift at δ3.6 ppm was mainly attributed to the absorption peak of the H in the methylene hydrogen of CH_2_-CH-CH_2_-. As shown in Figure 4, the number average and weight average molecular weight of the products synthesized with different monomer ratios are kept above 2.0 × 10^6^, which is much larger than the molecular weight of the homopolymer formed by the monomer in the synthesis reaction. The distribution is in the range of 1.1–1.3, the closer the value is to 1, the narrower the molecular weight distribution of the polymer. In combination with the infrared analysis, no double bonds were found to exist, indicating that the copolymerization was carried out successfully by AA, BA, and MMA with active double bonds.

### 3.1. Effect of Monomer Ratio on Emulsion

**Figure 5:**
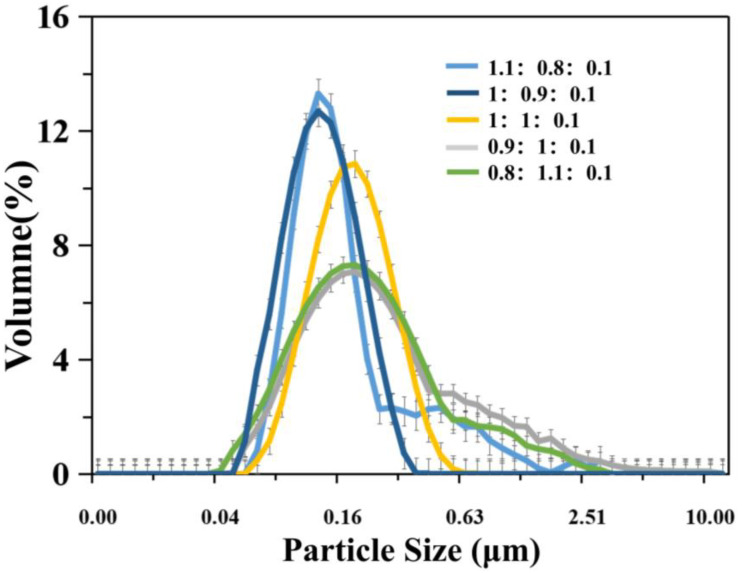
Article size distribution at different monomer ratios (butyl acrylate (BA)/methyl acrylate (MMA)/acrylic acid (AA)).

**Figure 6:**
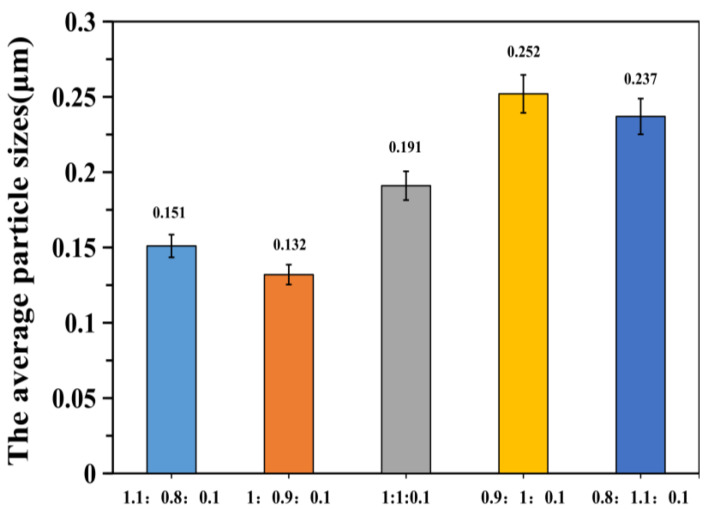
The average particle sizes of different monomer ratios (BA/MMA/AA).

**Figure 7:**
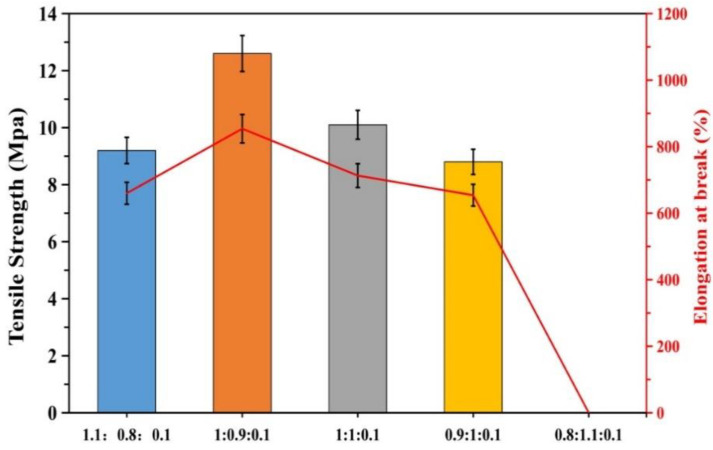
Mechanical properties of different monomer ratios (BA/MMA/AA).

**Figure 8:**
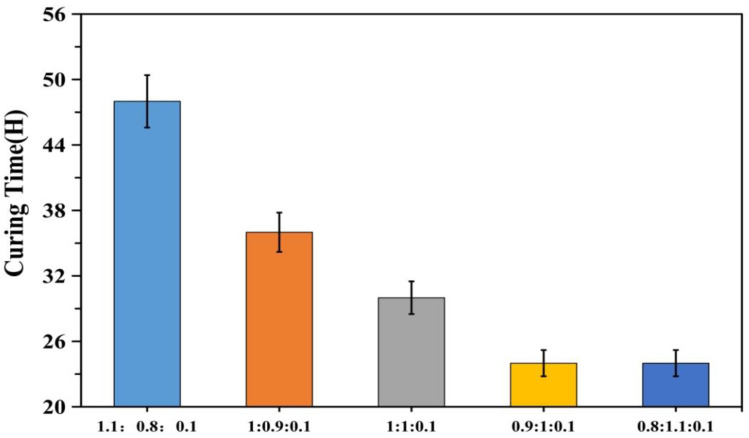
Curing time of different monomer ratios (BA/MMA/AA).

**Figure 9:**
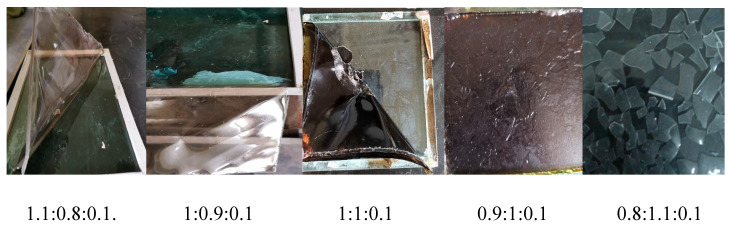
Forming properties of different monomers ratio (BA/MMA/AA).Visual inspection after film formation curing.

It is evident from Figure 5, Figure 6, Figure 7, Figure 8 and Figure 9, with the change in monomer ratios, that the decontaminant showed different film-forming effects after curing. As the proportion of BA in the component increased, the coating became soft, and the film-forming property increased, but became sticky after exceeding a certain proportion. From the structures of MMA and BA, it can be seen that the minimum film-forming temperature (MFT) of the BA monomer was much lower than that of the MMA monomer. In general, when the proportion of BA reached the continuous phase of the coating, the MFT of the coating was close to the MFT of BA, so it had a good film-forming property at room temperature. When the MMA ratio reached the continuous phase ratio in the film, the MFT of the film was close to the MMA-MFT, and its film-forming property was poor at room temperature. However, in order to reduce the film-forming temperature, the ratio of BA should not be too large, otherwise the film will become sticky.

Researchers suggest that the reason for this is in the process of film-coating formation, where the following steps will be carried out: solvent evaporation, random movement of latex particles, aggregation and accumulation of emulsion particles, and film solidification. Therefore, when the particle size was smaller, the heap gathered more closely, which is beneficial to the interpenetration and film formation between particles [40,41], for example, as shown in Figure 5, the log-normal distribution curve of particle size under different monomer ratios. In different monomer ratios, the size of the particle size and the distribution of the particle size range are different, and the corresponding film formation result of the coating also changes.

A good curing and film-forming ability is a prerequisite for the use of a decontaminant. Based on coating experiments on different monomer ratios, it can be seen that the test samples for BA/MMA/AA (1:0.9:0.1) were the best, as they formed continuous films on the surfaces and met the overall recovery requirements. Therefore, in the following study, this ratio of synthetic decontaminant was used as the standard for research.

### 3.2. Film-Forming Properties and Peelable Properties BA/MMA/AA (1:0.9:0.1)

**Figure 10:**
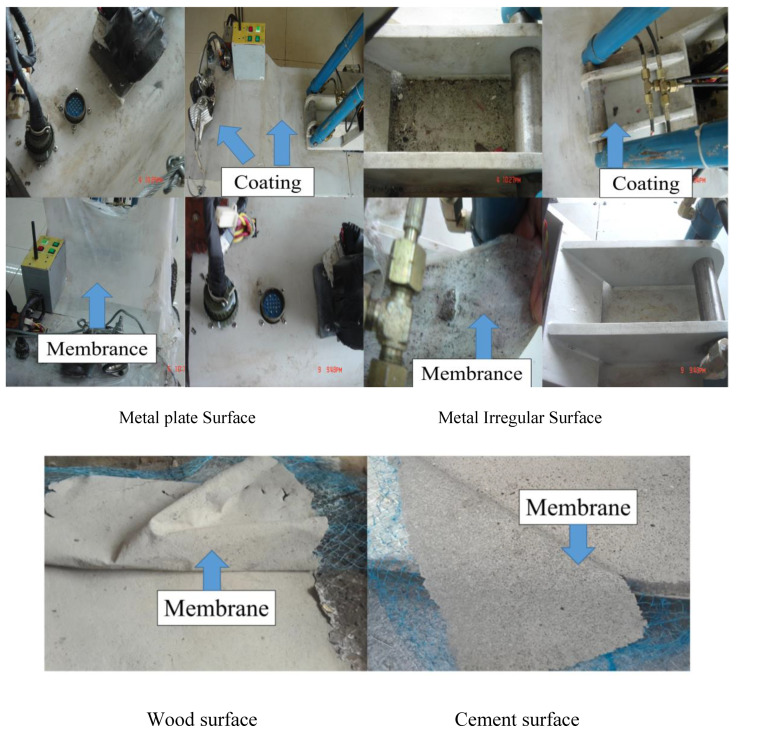
Examples of practical application BA/MMA/AA (1:0.9:0.1).

In order to verify the film-forming property and strippability of the synthesized detergent, the film-forming and strippability of the detergent after curing were investigated by coating the detergent on iron, wood, and cement floors. It can be seen from Figure 10 that the membrane was completely formed and separated at the selected experimental interface.

Good curing and film-forming abilities are significant factors influencing the use of decontaminants. At the same time, A moderate peel strength can ensure a good bonding ability, and it can quickly unseal. It can be seen in Table 2 that the test samples continuously formed films on different surfaces with complete peeling, and the peeling strength was moderate. 

### 3.3. Dust Capture and Surface Decontamination Performance of Polymer Substrate BA/MMA/AA (1:0.9:0.1)

The wettability of the polymer is also one of the major indicators affecting the decontamination performance. In the decontamination process, it is inevitable that large particles or substances that are loosely present on the surface of the object will be encountered. Wetting is used as a prerequisite for decontamination, which is a prerequisite for the smooth progress of the purification process; therefore, the wettability of the decontaminant should be studied.

**Figure 11:**
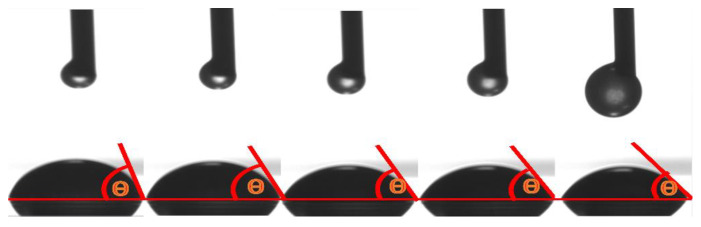
Contact angle of polymer substrate BA/MMA/AA (1:0.9:0.1).

**Figure 12:**
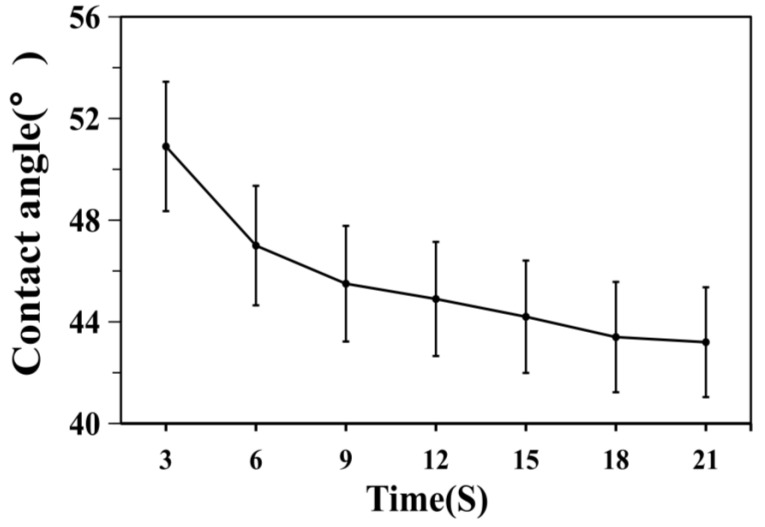
Contact angle data of polymer BA/MMA/AA (1:0.9:0.1).

**Figure 13:**
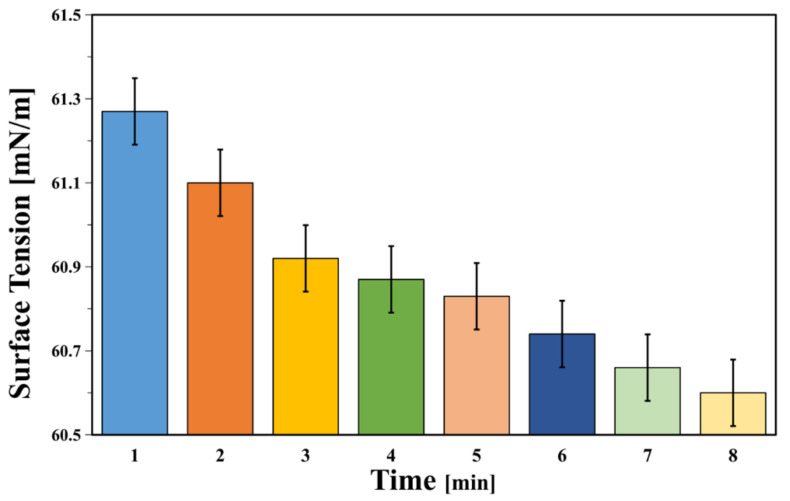
Tension of polymer BA/MMA/AA (1:0.9:0.1).

Figure 11 and Figure 12 indicate that the initial contact angle was 50.9°, and it showed a gradual decrease before eventually stabilizing over time. In addition, Figure 13 shows that as time progressed, the surface tension of the polymer was stable at 61.1 mN/m. The removal of contaminants from solid surfaces can be considered a capillary infiltration process by the decontaminant.

(2) $$\Delta P=\frac{\gamma_{1}COS\theta_{1}}{r}$$

From Formula (2), it can be inferred that a smaller contact angle and surface tension will be conducive to the process of capillary penetration; consequently, a better decontamination performance is expected because of the smaller contact angle and smaller surface tension in the polymer substrate. Therefore, obtaining a smaller contact angle and smaller surface tension are properties of great importance for the strippable decontaminant material.

Through the synthesis of raw decontaminant materials, and the preparation processes and the effects of the wetting analysis on the samples, we can see that, first, the decontaminant using water as a dispersing agent can wet the object effectively. Second, because of the existence of a hydrophobic microblock structure in the polymer, the hydrophobic groups in the microblock structure avoid contact with water and are adsorbed onto the gas–liquid surface of the solution. The water molecules in the solution exert less force on the hydrophobic groups on the surface of the solution, so the surface tension of the solution is rapidly reduced.

### 3.4. Solid Seal Performance BA/MMA/AA (1:0.9:0.1)

The solid seal performance is another important parameter for evaluating the decontamination efficiency. The sealing performance of the detergent was tested using Fe and Cu, as shown in Table 3 and Table 4. The sealing rate of all the sample areas was above 99%, and the average sealing rate reached 99.5%/99.6%. In addition, the H_2_ gas permeability of the polymer film is shown in Table 5.

#### Standard Deviation and Dispersion Measurements Regarding the Sealing Rate of BA/MMA/AA (1:0.9:0.1)

We calculated the standard deviation and dispersion degree of the paint sealing rate in different environments using Formulas (3) and (4) and the data in Table 6 and Table 7. The degree of dispersion of the data is calculated from the standard deviation of the data. From the data of Table 8 and Table 9, it can be seen that there was no large deviation in the tested data. The measured data were within the interval, proving that the data were authentic.

(3) $$\mu =\overset{-}{X}\pm Z_{\frac{2}{\partial }}\frac{\sigma }{\sqrt{n}}$$

(4) $$\sigma =\sqrt{\frac{\sum_{i=1}^{N}(x_{i}-\overset{-}{x)}}{N}}$$

As show in the Table 8 and Table 9,the results indicate that the detergent processes showed a good sealing performance and can meet the requirements of radioactive decontamination in practical applications. This may be attributed to two main factors, namely: (1) the molecular chain of the prepared terpolymer is rigid, or (2) the prepared polymer has a relatively regular structure and good crystallinity; therefore, the diffusion of small molecules in the amorphous region of the polymer is limited. Similar results were found by Mittal K.L [42], who showed that a polymer with a relatively regular structure enhanced the polymer seal performance.

## 4. Conclusions

In this paper, a kind of ternary system was prepared by pre-emulsion polymerization with butyl acrylate, methyl methacrylate, acrylic acid as the reactive monomer, sodium dodecyl sulfate as the active agent, potassium persulfate as the initiator, and water as the dispersion medium. The Fourier-transform infrared (FTIR) spectroscopy, nuclear magnetic resonance (^1^H-NMR), ICP emission spectrometer, surface tension tester, and universal testing machine were used to characterize the structure and the morphology of the composite materials. The factors affecting the decontamination performance of the stripping decontaminant were studied: the results show the use of acrylic esters as monomers and the use of pre-emulsification polymerization can achieve a suitable particle size and a relatively small particle size distribution. It can effectively promote film formation and form a film body with a good sealing performance. Based on the coating experiments on the different monomer ratios, the test samples with the ratio of BA/MMA/AA at 1:0.9:0.1 are the best. The membrane has good mechanical properties and sealing performances, moderate peel strength—the tensile strength was 12.8 Mpa and elongation at break was 840%—and can be completely peeled off after application on different surfaces. The prepared decontamination coating can be used to remove loose contaminants from a large area of surface because it can play a fixed role in pollutants. In the experiment, the sealing rate of metals on different surfaces was above 90%, which can meet the needs of equipment that needs to continue to be used in the polluted environment.

It was found through experiments that when the coating has a good wettability, it can be brought into close contact with the pollutants. The smaller particle size can help the coating to form a film, and it can better allow the coating to penetrate into the pores formed between the surface of the object and the pollutant while improving the removal efficiency of the pollutant and avoiding secondary pollution. Therefore, the particle size will affect the process of suppressing and collecting pollutants, and should be controlled during the polymerization of the monomer in order to obtain a better particle size and distribution. At the same time, it is necessary to consider the selection of synthetic monomers and additives. For example, acrylates as hydrophobic monomers can reduce the surface tension, so that the coating has better wettability and lower surface tension, which can facilitate the decontamination process.

## Figures and Tables

**Table 1:** The monomer commonly used in acrylic polymer emulsions.

Monomer			Boiling Point (°C/mmHg)	Specific Gravity (D)	Refractive Index	Tg (k)
Type	Name	English Abbreviation				
Soft monomer (flexibility)	Butyl acrylate	BA	35/8	0.8998	1.4190	219.15
	Ethyl acrylate	EA	43/103	0.9234	1.4068	251.15
	Octyl acrylic ester	OA	—	—	—	—
	Isooctyl acrylate	2-EHA	85/5	0.8852	1.4365	—
	Isooctyl methacrylate	2-EHMA	47/0.1	—	1.4380	—
	Dodecane methacrylate	LMA	—	—	—	—
Hard monomer (hardness)	Methyl acrylate	MA	80	09524	1.4040	281.15
	Methyl methacrylate	MMA	100	0.939	1.4120	373.15
	Ethyl methacrylate	EMA	119	0.909	1.4116	338.15
	N-butyl methacrylate	BMA	98/90	0.889	1.4220	293.15
	Vinyl acetate	VAc	72-73	0.932		303.15
	Styrene	ST	145-6	0.909	1.4116	373.15
Functional monomer (abrasion resistance, cross-linking degree, water resistance, etc.)	Acrylonitrile	AN	77.3	0.806	13888	369.15
	Acrylic acid	AA	141	1.045	1.4185	379.15
	Methacrylate	MAA	160	1.015	1.4288	403.15
	Acrylamide	AM	12.5/25	1.122	1.460	426.15
	N-carboxyacrylamide	N-MAM	75	1.185	—	—
	Hydroxyethyl acrylate	2-HEA	82/5	1.1038	1.4505	—
	Hydroxypropyl acrylate	2-HPA	77/5	1.056	1.4448	—
	Carboxypropyl methacrylate	2-HPMA	96/10	1.027	1.4460	346.15
	Glycidyl acrylate	GA	57/2	1.1074	1.4460	—
	Glycidyl methacrylate	GMA	189	1.073	1.4482	—
	Ethylene glycol dimethacrylate	—	97/4	1.051	1.4520	—
	Dimethacrylic acid-glycol ester		162/2	1.071	1.4570	—
	Itaconic acid	IA	resolve	1.632	—	—
	Maleic acid	NA	139–140	1.609	—	—
	Maleic anhydride	NAA	60	1341	—	—

**Table 2:** Film formation and peelable properties of polymer substrate BA/MMA/AA (1:0.9:0.1).

Sample Test Environment		Mean Peel Strength (N.M^−1^)	Film Forming Property	Stripping Degree
Wood Surface		0.570	Continuous film	100%
Metal plate	Flat Surface	0.873	Continuous film	100%
	Irregular Surface	0.852	Continuous film	100%
Cement flat surface		0.653	Continuous film	100%

**Table 3:** Sealing rate of concave and convex parts of BA/MMA/AA (1:0.9:0.1).

	Samples	1	2	3	Average
Solid seal before	Fe^3+^/(mg/L)	117.5	72.9	143.0	111.1
	Fe/(mg/dm^2^)	23.5	14.6	28.6	22.2
	Cu^2+^/(mg/L)	18.9	28.9	51.1	33.0
	Cu/(mg/dm^2^)	3.8	5.8	10.2	6.6
Solid seal after	Fe^3+^/(mg/L)	2.6	6.0	6.9	5.2
	Fe/(mg/dm^2^)	0.5	1.2	1.4	1.0
	Cu^2+^/(mg/L)	1.4	0.9	0.9	1.1
	Cu/(mg/dm^2^)	0.3	0.2	0.2	0.2
Enclosed rate	Fe (%)	99.8	99.2	99.5	99.5
	Cu (%)	99.2	99.7	99.9	99.6

**Table 4:** Sealing ratio of the vertical surface of BA/MMA/AA (1:0.9:0.1).

	Samples	Left Side 1	Left Side 2	Right Side 3	Right Side 4	Average
Solid seal before	Fe^3+^/(mg/L)	57.1	117.7	327.6	78.5	145.2
	Fe/(mg/dm^2^)	11.4	23.5	65.5	15.7	29.0
	Cu^2+^/(mg/L)	17.8	18.3	37.4	18.3	22.9
	Cu/(mg/dm^2^)	3.6	3.7	7.5	3.7	4.6
Solid seal after	Fe^3+^/(mg/L)	1.9	1.8	17.4	2.9	6.0
	Fe/(mg/dm^2^)	0.4	0.4	3.5	0.6	1.2
	Cu^2+^/(mg/L)	1.2	0.9	1.8	0.6	1.1
	Cu/(mg/dm^2^)	0.2	0.2	0.4	0.1	0.2
Enclosed rate	Fe (%)	99.7	99.8	99.5	99.6	99.7
	Cu (%)	99.4	99.5	99.5	99.9	99.6

**Table 5:** Gas permeability of polymer substrate film BA/MMA/AA (1:0.9:0.1).

Samples	Sample Thickness/mm	Permeability/(cm^3^.cm/cm^2^ d.bar)
1	0.30	0.0070
2	0.30	0.0069
3	0.30	0.0072
4	0.30	0.0069

**Table 6:** Sealing rate of concave and convex parts of BA/MMA/AA (1:0.9:0.1).

	Samples	1	2	3	Average
Enclosed rate	Fe (%)	99.8	99.2	99.5	99.5
	Cu (%)	99.2	99.7	99.9	99.6

**Table 7:** Sealing ratio of the vertical surface of BA/MMA/AA (1:0.9:0.1).

	Samples	Left Side 1	Left Side 2	Right Side 3	Right Side 4	Average
Enclosed rate	Fe (%)	99.7	99.8	99.5	99.6	99.7
	Cu (%)	99.4	99.5	99.5	99.9	99.6

**Table 8:** Deviation and dispersion measurements regarding the sealing rate of the concave and convex parts of BA/MMA/AA (1:0.9:0.1).

Sample	σ (Standard Deviation)	μ (*p* = 95%) Estimation Error	
Fe (%)	0.339	99.161	99.839
Cu (%)	0.409	99.91	100.09

**Table 9:** Deviation and dispersion measurements regarding the sealing ratio of the vertical surface of BA/MMA/AA (1:0.9:0.1).

Sample	σ (Standard Deviation)	μ (*p* = 95%) Estimation Error	
Fe (%)	0.126	99.524	99.810
Cu (%)	0.218	99.357	99.793
